# Prevalence of Cigarette Smoking and Associated Factors among Residents of Hossana Town, Southern Ethiopia

**DOI:** 10.1155/2022/2272281

**Published:** 2022-01-20

**Authors:** Abera Beyamo Mekiso, Temesgen Tamirat Fonkamo, Tekle Ejajo Wontamo, Fitsum Endale Liben, Ermias Abera Turuse, Aregash Mecha Watumo, Lonsako Abute Woiloro, Dawit Sullamo Erjino, Tegegn Tadesse Arficho, Dejene Ermias Mekengo

**Affiliations:** School of Public Health, College of Medicine and Health Sciences, Wachemo University, Hosaena, Ethiopia

## Abstract

**Background:**

Tobacco is the only legal product that kills a large number of its consumers when used as intended by producers. Information on cigarette smoking and associated factors among adults at the household level is very limited.

**Objective:**

To assess prevalence of cigarette smoking and associated factors among residents of Hossana town, Hadiya zone, Southern Ethiopia, 2020.

**Methods and Materials:**

A cross-sectional study design was performed. A structured questionnaire was used to collect data. Bivariate and multivariable binary logistic regression was used to identify risk factors of cigarette smoking. Variables significant at a *p* value of less than 0.05 were considered as independent predictors. Hosmer and Lemeshow test statistics were done to test the model fitness for the final model. Similarly, multicollinearity was checked by using collinearity statistics (tolerance and VIf).

**Result:**

In total, 591 people responded to the survey, resulting in a 98.2% response rate. Among the study participants, cigarette smokers were 183 (31.0%). Educational status, alcohol use, and parental smoking were all found to have a significant relationship with cigarette smoking among research participants in Hosanna town. When compared to people with a college education or above, illiterates are approximately nine times more likely to consume cigarettes (95% CI = 9.058 (3.52, 22.469)). Alcoholics are about twice as likely as nondrinkers to smoke cigarettes (95% CI = 2.288 (1.548, 3.383)). Those who have cigarette-smoking parents are approximately twice as likely as their counterparts to smoke cigarettes (95% CI = 2.288 (1.548, 3.383)).

**Conclusion:**

According to this survey, the prevalence of cigarette smoking was high. Furthermore, cigarette smoking was linked to illiteracy, alcohol consumption, and parental smoking in this study.

## 1. Background

When used as intended by makers, tobacco is the only allowed product that kills a considerable number of its customers. Tobacco is available in both smoked and nonsmoked forms. Tobacco is smoked in a variety of ways, including cigarettes (made or hand-rolled), cigars, pipes, and water pipes. Manufactured cigarettes are the most extensively used smoked tobacco product on the globe [1, 2].

Tobacco usage is linked to six of the world's eight main causes of mortality. Tobacco use causes lung cancer, laryngeal cancer, kidney cancer, bladder cancer, stomach cancer, colon cancer, oral cancer, and esophageal cancer, as well as leukemia, chronic bronchitis, chronic obstructive pulmonary disease, ischemic heart disease, stroke, miscarriage and premature birth, birth defects, and infertility [1, 3].

Tobacco continues to be a global health pandemic, killing around 6 million people each year and incurring hundreds of billions of dollars in annual financial losses [4].

Tobacco smoke contains about 7000 synthetic chemicals and substances. Hundreds are hazardous, and more than 70 cause diseases such as cancer. Exposure to smokers (friends, parents, and instructors), nicotine availability, low socioeconomic level, poor academic achievement, low self-esteem, and a lack of skill to prevent tobacco use are all factors linked to teenage cigarette smoking [5–7].

In 2013, 22 percent of the world's population aged 15 and up, including 36 percent of men and 8% of women [7], were predicted to smoke cigarettes, and by 2025, approximately 1.6 billion people are expected to be cigarette smokers [8]. Women in upper middle and higher income countries smoke more cigarettes than women in poor and lower-middle income countries [9].

The majority of smokers (89%) start smoking before they reach the age of 19, when they are still living with their parents [5, 10–12]. The findings reveal that starting to smoke at a younger age is linked to smoking more cigarettes per day later in life than starting at an older age, implying that postponing the initiation of smoking may affect the chance of becoming addicted to cigarettes [13]. There are many potential environmental exposure sites for cigarette smoking: public places, retail shops, and smoking in cars and home. Among which home was identified as the potential environmental exposure site (second hand smoking) to children and adults as well as potential for youth's initiation [14–17].

The Framework Convention on Tobacco Control (WHO FCTC) of the World Health Organization recognizes the significant impact of tobacco use and the urgent need to avoid it [4, 18]. In contrast to past drug control treaties, the WHO FCTC is an evidence-based treaty that aids in the development of a regulatory plan to handle addictive substances. It highlights the relevance of demand and supply reduction methods. [18]

But according to the investigators knowledge, information on prevalence and factors that influence it is extremely rare in low and middle income countries, particularly Ethiopia. As a result, the purpose of this study is to determine the prevalence of cigarette smoking in Hossana, Ethiopia, as well as the factors that influence it.

## 2. Methods

### 2.1. Study Design and Area

A community-based cross sectional study design was conducted among residents in Hadiya zone, Hossana town, from April 01 to 30, 2020. Hossana town is located at 230 km South of Addis Ababa. According to the 2007 national census, the projected total population of the town is 108,428 (53,129 males ad 55,299 females). The total number of HHs in Bobicho Kebele was 5790 and in Jelo Naremo Kebele was 5490 where actual data was collected.

### 2.2. Study Population and Sampling

The sample size was calculated using a one population proportion formula, considering 57% [19] proportion of individuals who smoke cigarette, 5% margin of error and correction formula, and 5% estimated nonresponse rate and 1.5 design effect. A multistage sampling technique was employed. From a total of 6 kebele in Hossana town (Lich-amba Kebele, Arada Kebele, Heto Kebele, J/Naremo Kebele, Bobicho Kebele, and Sech duna Kebele), 2 kebeles (Jelo/Naremo Kebele and Bobicho Kebele) were selected randomly using a lottery technique. The households were selected by systematic random sampling after determination of the *K*th interval for each kebeles (Bobicho *k* = 10, Jelo/Naremo = 9). To select the starting household, a pen was pinned then households in the direction of the tip of the pen were selected. Finally, the starting household was selected randomly from the first *K*th households in the direction of the tip of the pen after coding the first households 1 up to *K* (1-10 for Bobicho and 1-9 for Jelo Naremo). The randomly selected household's number was 3 and 7 for Bobicho and Jelo/Naremo Kebeles, respectively. Then, households were selected by jumping every *K*th interval. Eventually, the head of the selected household was interviewed, but in case when the household was headed by both husband and wife, female head was selected. In the absence of heads of household, the adults with age ≥ 18 years were interviewed.

### 2.3. Data Collection Instrument, Data Collectors, and Data Quality Control

Data was collected using an interviewer-administered structured questionnaire. The instrument was first modified in English from earlier research conducted in the Amhara region [19] and Southern Ethiopia [20] before being translated into Hadiyisa and Amharic by language experts in Hadiyisa and Amharic and then returned to English by other language experts to guarantee consistency. The questionnaire is composed of socio demographic characteristics, behaviour-related characteristics, and environment related characteristics of respondent. Eight diploma nurses as data collectors and four BSc in health as supervisors were employed. Data collectors and supervisors received two days of training from the lead investigator to assure the quality of the field operation. The supervisors had overseen the data collection procedure on a daily basis and performed quality checks during the data collection. To ensure the tool's dependability, it was pretested on 5% of the sample before the real data collection days in Gibe Woreda, which is 30 kilometers away from the study area. The questionnaire was not changed based on the findings and input gathered during the pretesting procedure. The main data analysis did not contain the pretested data. During the data collection process, participants were able to choose acceptable locations.

### 2.4. Data Processing and Analysis

Before entering the data into the software, all of the data was carefully validated. The data was then entered into the Epi Data version 3.1 software on a computer. The software was built with data types and sizes in mind, as well as categories, validating permissible values and ranges, and codes for missing values. For each of the variables, descriptive analysis was used to verify frequency, distribution, and missing values. To see if there was a direct link between cigarette smoking and the independent variables, bivariate analysis was used. The Chi-square test was used to see if the variables met the assumptions. To find the factors that affect the prevalence of cigarette smoking, a variable with a *p* value of 0.25 on bivariate analysis was incorporated into multivariable logistic regression. The degrees of relationship between the independent variable and cigarette smoking status were quantified using the odds ratio and 95 percent confidence intervals. The results with a *p* value of less than 0.05 were declared statistically significant, while the remainder was disproved. The multicollinearity diagnostic test VIF in linear regression was used to assess for collinearity among independently related variables, and none were found to be collinear.

## 3. Result

### 3.1. Socio-Demographic-Related Characteristics

Overall 591 participants were participated in this study which makes a response rate of 93.8%. From study participants, 261 (44.2%) were 18-27 years old, 504 (85.3%) of the participants were males, 408 (69.0%) were married, 347 (58.7%) have 4-6 family size, 269 (45.5%) were protestant, 251 (42.5%) were Hadiya, 217 (36.7%) were daily laborer, 371 (62.8%) were ≤12 grade educational status, and 155 (26.2%) have 3001-4500 monthly income (Table 1).

### 3.2. Prevalence of Cigarette Smoking

**Figure 1:**
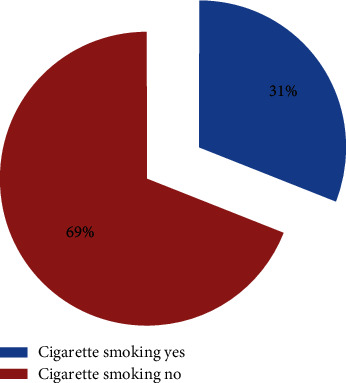
Prevalence of cigarette smoking among study participants in Hosanna town, Southern Ethiopia, 2019/20.

From the study participants, 183 (31.0%) were cigarette smokers (see Figure 1).

### 3.3. Behaviour- and Environment-Related Characteristics

From study participants, 280 (47.4%) were Khat chewers, 339 (57.4%) were alcohol drinkers, 317 (53.6%) were having cigarette smoker parents, 306 (51.8%) were having cigarette smoking siblings, and 366 (61.9%) were having cigarette smoking peers (Table 2).

### 3.4. Bivariable Analysis of Factors Linked to Cigarette Smoking

Sex, family size, occupation, educational status, monthly income, khat chewing, alcohol use, parental cigarette smoking, siblings' cigarette smoking, peers' cigarette smoking, and cigarette accessibility were all eligible for multivariable analysis (Table 3).

### 3.5. Factors Associated with Cigarette Smoking in Multivariable Logistic Regression

Educational status, alcohol use, and parental smoking were all found to have a significant relationship with cigarette smoking among research participants in Hosanna town. This finding revealed that illiterates are nearly nine times AOR at 95% CI = 9.058 (3.652, 22.469) smoke cigarette when compared to individuals with college and above. Alcohol drinkers are nearly two times AOR at 95% CI = 2.288 (1.548, 3.383) smoke cigarette than their counter parts. Those individuals having cigarette smoking parents are nearly two times AOR at 95% CI = 2.288 (1.548, 3.383) smoke cigarette than their counter parts (Table 4).

## 4. Discussion

The overall prevalence of cigarette smoking was 31.0 percent, according to the result of this research. This study's prevalence is similar to that of studies conducted in China (31.8%) [21] and Tunisia (30.4%) [22]. This result was higher than those of studies conducted in Eastern Ethiopia (28%) [23], Madagascar (28.5%) [24], Bangladesh (23.19%) [25], and Misrak Badewacho (23.6%) [20]. This result was lower than those found in Jimma Town (35.5%) [26] and Amhara area (57.0%) studies [19]. This disparity could be attributable to socioeconomic and cultural inequalities, as well as variances in study settings. In this study, the prevalence of cigarette smoking is higher among males than females, among drinkers against nondrinkers, among those with smoking peers versus nonsmokers, and among those with cigarette access versus nonsmokers.

In this study, educational status, particularly illiteracy, was found to be substantially related with cigarette smoking. Various conclusions have been published in earlier studies regarding the relationship between educational status and cigarette smoking. This finding was supported with previously conducted in Uttar Pradesh [27], Jimma [28], Helaba [16], and low and middle income countries [21, 29, 30]. It is possible that the link between illiteracy and cigarette smoking is due to a lack of knowledge regarding tobacco's harmful effects on health.

This study finding revealed that alcohol drinking has significant association with cigarette smoking. This finding was supported with previously conducted in Nigeria [31]. This observed association might be due to the consumption of alcohol that also triggers the probability of having cigarette smoking.

Also, this study finding revealed that those having parental cigarette smoking have significant association with cigarette smoking. This finding has been supported by study conducted in China [32–34] and in Sudan [35]. The possible reason for this observed association could be taking parents as their role model and passing extended time and intact contact with parents.

## 5. Conclusion and Recommendation

The prevalence of cigarette smoking among residents in the Hossana town is high. Furthermore, cigarette smoking was found to be substantially linked to illiteracy, alcohol consumption, and parental smoking in this study. The zonal health department, woreda health office, and health facilities should focus on regular surveys on prevalence and determinants of all forms of tobacco use in the general population and adjusting outreach session program for health professionals and health extension workers as much as possible. The researchers are recommended to conduct further research on cigarette smoking with strong epidemiological design to overcome the limitation of this study.

## 6. Strength and Limitations

This study looks into the prevalence of cigarette smoking and related factors in adult age groups that are rarely looked into. Because this study is based on self-report, the prevalence of cigarette smoking may be under- or overreported.

## Figures and Tables

**Table 1:** Socio-demographic characteristics of study participants in Hossana town, 2019/2020.

Variable	Category	Number	Percent (%)
Age	18-27 years	261	44.2
	28-37 years	161	27.2
	38-47 years	163	27.6
	≥48	6	1.0
Sex	Male	504	85.3
	Female	87	14.7
Marital status	Single	165	27.9
	Married	408	69.0
	Divorced	7	1.2
	Widowed	11	1.9
Family size	1-3	165	27.9
	4-6	347	58.7
	7-8	7.6	7.6
	≥8	5.8	5.8
Religion	Protestant	269	45.5
	Orthodox	245	41.5
	Muslim	47	8.0
	Catholic	30	5.1
Ethnicity	Hadiya	251	42.5
	Kembata	191	32.3
	Gurage	70	11.8
	Silte	44	7.4
	Amhara	16	2.7
	Wolaita	19	3.2
Occupation	Daily laborer	217	36.7
	Gov't employed	208	35.2
	Self-employed	72	12.5
	Student	6	1
	Housewife	5	0.8
	No job	3	0.5
	Merchant	80	13.5
Educational status	Illiterate	161	27.2
	≤12 grade	371	62.8
	≥College	59	10.0
Monthly income	0-1500	83	14.0
	1501-3000	86	14.6
	3001-4500	155	26.2
	4501-6000	88	14.9
	≥6001	179	30.3

**Table 2:** Behaviour- and environment-related characteristics of study participants in Hosanna town, 2019/2020.

Variables	Categories	Number	Percent (%)
Chat chewing	Yes	280	47.4
	No	311	52.6
Alcohol consumption	Yes	339	57.4
	No	252	42.6
Is your parents smoke cigarette	Yes	317	53.6
	No	274	46.4
Siblings smoke cigarette	Yes	306	51.8
	No	285	48.2
Peers smoke cigarette	Yes	366	61.9
	No	225	38.1
Who is the role model for your cigarette smoking	Father	71	30.5
	Siblings	67	28.8
	Peers	95	40.8
Having close attachment with your families	Yes	339	57.4
	No	252	42.6
During the past 30 days, did someone smoke in closed areas in your working environment	Yes	339	57.4
	No	252	42.6
Cigarette accessibility	Yes	380	64.3
	No	211	35.7

**Table 3:** Bivariable analysis of factors associated with cigarette smoking among residents in Hossana town, 2019/2020.

Variable	Category	Cigarette smoking		COR (95% CI)	*p* value
		Yes	No		
Sex	Male	168 (28.4%)	336 (56.9%)	8.2 (0.030, 0.191)	<0.001
	Female	5 (0.8%)	82 (13.9%)	1	
Family size	1-3	8 (1.4%)	157 (26.6%)	0.082 (0.031, 0.222)	<0.001
	4-6	139 (33.0%)	208 (25.7%)	2.072 (1.005, 4.272)	0.048
	7-8	13 (2.2%)	32 (5.4%)	0.656 (0.255, 1.689)	0.383
	≥8	13 (2.2%)	21 (3.6%)	1	
Occupation	Daily laborer	60 (10.2%)	157 (26.6%)	1	
	Gov't employed	60 (10.2%)	148 (25.0%)	3.299 (2.202, 4.943)	<0.001
	Self-employed	19 (3.2%)	53 (9.0%)	0.938 (0.513, 1.714)	0.835
	Merchant	31 (5.2%)	49 (8.3%)	1.655 (0.965, 2.839)	0.067
	Other	3 (0.5%)	11 (1.9%)	0.714 (0.192, 2.647)	0.614
Educational status	Illiterate	8 (1.4%)	153 (25.9%)	0.071 (0.030, 0.171)	<0.001
	≤12 grade	140 (23.7%)	231 (39.1%)	1.523 (0.874, 2.654)	0.137
	≥College	25 (4.2%)	34 (5.8%)	1	
Monthly income	0-1500	55 (9.3%)	28 (4.7%)	1	
	1501-3000	6 (1.0%)	79 (13.4%)	0.039 (0.015, 0.100)	<0.001
	3001-4500	74 (12.5%)	82 (13.9%)	0.459 (0.264, 0.799)	0.006
	4501-6000	14 (2.4%)	74 (12.5%)	0.096 (0.046, 0.200)	<0.001
	≥ 6001	24 (4.1%)	155 (26.2%)	0.411 (0.239, 0.707)	0.001
Khat chewing	Yes	81 (13.7%)	258 (43.7%)	0.221 (0.155, 0.314)	<0.001
	No	92 (15.6%)	160 (27.1%)	1	
Alcohol consumption	Yes	81 (13.7%)	258 (43.7%)	1	
	No	92 (15.6%)	160 (27.1%)	4.533 (3.182, 6.457)	<0.001
Is your parents smoke cigarette	Yes	72 (12.2%)	245 (41.5%)	1	
	No	101 (17.1%)	173 (29.3%)	4.565 (3.200, 6.515)	<0.001
Siblings smoke cigarette	Yes	73 (12.4%)	233 (39.4%)	1	
	No	156 (26.4%)	129 (21.8%)	3.860 (2.716, 5.485)	<0.001
Peers smoke cigarette	Yes	95 (16.1%)	271 (45.9%)	1	
	No	78 (13.2%)	147 (24.9%)	4.201 (2.948, 5.986)	<0.001
Cigarette accessibility	Yes	102 (17.3%)	278 (47.0%)	1	
	No	71 (12.0%)	140 (23.7%)	4.121 (2.883, 5.889)	<0.001

**Table 4:** Multivariable analysis of factors associated with cigarette smoking among study participants in Hossana town, 2019/20.

Variable	Category	Cigarette smoking		COR (95% CI)	AOR (95% CI)	*p* value
		Yes	No			
Family size	1-3	8 (1.4%)	157 (26.6%)	0.082 (0.031, 0.222)	0.873 (0.912, 6.139)	0.645
	4-6	139 (33.0%)	208 (25.7%)	2.072 (1.005, 4.272)	0.944 (0.413, 2.161)	0.892
	7-8	13 (2.2%)	32 (5.4%)	0.656 (0.255, 1.689)	0.644 (0.233, 1.782)	0.392
	≥8	13 (2.2%)	21 (3.6%)	1	1	
Occupation	Daily laborer	60 (10.2%)	157 (26.6%)	1	1	
	Gov't employed	60 (10.2%)	148 (25.0%)	3.299 (2.202, 4.943)	0.017 (0.005, 8.153)	0.567
	Self-employed	19 (3.2%)	53 (9.0%)	0.938 (0.513, 1.714)	0.028 (0.009, 3.184)	0.312
	Merchant	31 (5.2%)	49 (8.3%)	1.655 (0.965, 2.839)	0.007 (0.001, 1.039)	0.154
	Other	3 (0.5%)	11 (1.9%)	0.714 (0.192, 2.647)	0.017 (0.006, 2.051)	0.241
Monthly income	0-1500	55 (9.3%)	28 (4.7%)	1	1	
	1501-3000	6 (1.0%)	79 (13.4%)	0.039 (0.015, 0.100)	1.224 (0.881, 17.533)	0.432
	3001-4500	74 (12.5%)	82 (13.9%)	0.459 (0.264, 0.799)	0.331 (0.664, 1.311)	0.231
	4501-6000	14 (2.4%)	74 (12.5%)	0.096 (0.046, 0.200)	0.977 (0.564, 2.332)	0.114
	≥ 6001	24 (4.1%)	155 (26.2%)	0.411 (0.239, 0.707)	0.675 (0.977, 1.871)	0.224
Educational status	Illiterate	8 (1.4%)	153 (25.9%)	0.071 (0.030, 0.171)	9.058 (3.652, 22.469)	<0.001^∗∗^
	≤12 grade	140 (23.7%)	231 (39.1%)	1.523 (0.874, 2.654)	0.646 (0,367, 1.139)	0.131
	≥ College	25 (4.2%)	34 (5.8%)	1	1	
Chat chewing	Yes	81 (13.7%)	258 (43.7%)	0.221 (0.155, 0.314)	0.664 (0.222, 1.986)	0.464
	No	92 (15.6%)	160 (27.1%)	1	1	
Is your parents smoke cigarette	Yes	72 (12.2%)	245 (41.5%)	1	1	
	No	157 (26.6%)	117 (19.8%)	4.565 (3.200, 6.515)	2.288 (1.548, 3.383)	<0.001^∗∗^
Siblings smoke cigarette	Yes	73 (12.4%)	233 (39.4%)	1	1	
	No	156 (26.4%)	129 (21.8%)	3.860 (2.716, 5.485)	0.847 (0.339, 2.118)	0.722
Peers smoke cigarette	Yes	95 (16.1%)	271 (45.9%)	1		
	No	134 (22.7%)	91 (15.4%)	4.201 (2.948, 5.986)	1.472 (0.658, 3.297)	0.347
Cigarette accessibility	Yes	102 (17.3%)	278 (47.0%)	1	1	
	No	127 (60.2%)	84 (14.2%)	4.121 (2.883, 5.889)	2.354 (0.117, 5.980)	0.921
Alcohol consumption	Yes	81 (13.7%)	258 (43.7%)	4.533 (3.182, 6.457)	2.320 (1.331, 8.440)	0.004^∗^
	No	148 (25.0%)	104 (17.6%)	1	1	

## Data Availability

The data that supports the findings of this study are available; however, due to some sensitive problems, there may be some restrictions on their use. However, upon reasonable request, data can be obtained from the respective author.

## Abbreviations

AOR: Adjusted odds ratio
COR: Crude odds ratio
FCTC: Framework Convention on Tobacco Control
SPSS: Statistical Package for the Social Sciences
WHO: World Health Organization.
